# Recent Advances in the Molecular Effects of Biostimulants in Plants: An Overview

**DOI:** 10.3390/biom11081096

**Published:** 2021-07-25

**Authors:** Miguel Baltazar, Sofia Correia, Kieran J. Guinan, Neerakkal Sujeeth, Radek Bragança, Berta Gonçalves

**Affiliations:** 1 Centre for the Research and Technology of Agro-Environmental and Biological Sciences (CITAB), University of Trás-os-Montes and Alto Douro, 5000-801 Vila Real, Portugal; sofiammcorreia@gmail.com (S.C.); bertag@utad.pt (B.G.); 2 Institute for Innovation, Capacity Building and Sustainability of Agri-Food Production (Inov4Agro), University of Trás-os-Montes and Alto Douro, 5000-801 Vila Real, Portugal; 3 BioAtlantis Ltd., Clash Industrial Estate, Tralee, V92 RWV5 County Kerry, Ireland; research@bioatlantis.com (K.J.G.); Plant.Research@BioAtlantis.com (N.S.); 4 BioComposites Centre, Bangor University, Bangor LL57 2UW, UK; r.braganca@bangor.ac.uk

**Keywords:** biostimulant, gene expression, humic substances, protein hydrolysates, seaweed extracts, microorganisms

## Abstract

As the world develops and population increases, so too does the demand for higher agricultural output with lower resources. Plant biostimulants appear to be one of the more prominent sustainable solutions, given their natural origin and their potential to substitute conventional methods in agriculture. Classified based on their source rather than constitution, biostimulants such as humic substances (HS), protein hydrolysates (PHs), seaweed extracts (SWE) and microorganisms have a proven potential in improving plant growth, increasing crop production and quality, as well as ameliorating stress effects. However, the multi-molecular nature and varying composition of commercially available biostimulants presents challenges when attempting to elucidate their underlying mechanisms. While most research has focused on the broad effects of biostimulants in crops, recent studies at the molecular level have started to unravel the pathways triggered by certain products at the cellular and gene level. Understanding the molecular influences involved could lead to further refinement of these treatments. This review comprises the most recent findings regarding the use of biostimulants in plants, with particular focus on reports of their molecular influence.

## 1. Introduction

As the world population increases, higher demands will be placed on the agricultural sector to enhance production, yield and throughput. Increasing crop yield as we reach the limits in the genetic potential of staple crops, as well as the decrease in area of arable land, demands more production using less resources. This is usually achieved through the use of chemical fertilizers and/or pesticides, ameliorating the afore mentioned factors and the those of biotic and abiotic stresses [1]. However, indiscriminate use of agrochemicals has long lasting consequences on the environment, with some even being prohibited from further use, making agriculture one of the main sources of nonpoint pollution [2]. As the scientific community calls for more sustainability and environmentally friendly systems in agricultural practices, research on natural resources as alternatives to traditional chemical methods, such as plant biostimulants extracts, has been increasing in recent years [3].

Biostimulants are becoming more prominent in terms of economic value. The European Biostimulant Industry Council (EBIC) estimates a market range of around 1.5 to 2 billion USD in 2022 and a compound annual growth rate of around 10 to 12% [4]. However, what exactly is the definition of a plant biostimulant? According to EU regulation, a plant biostimulant is “a product stimulating plant nutrition processes independently of the product’s nutrient content with the sole aim of improving one or more of the following characteristics of the plant or the plant rhizosphere: (a) nutrient use efficiency; (b) tolerance to abiotic stress; (c) quality traits; (d) availability of confined nutrients in soil or rhizosphere” [5]. One of the most widely accepted scientific definitions of a plant biostimulant was proposed by du Jardin [6] in 2015: “A plant biostimulant is any substance or microorganism applied to plants with the aim to enhance nutrition efficiency, abiotic stress tolerance and/or crop quality traits, regardless of its nutrients content”. However, as Yakhin et al. [7] pointed out in their extensive review, no previous definition is complete, and instead proposed the following: “A biostimulant is a formulated product of biological origin that improves plant productivity as a consequence of the novel, or emergent properties of the complex of constituents, and not as a sole consequence of the presence of known essential plant nutrients, plant growth regulators, or plant protective compounds” [7]. In summary, a biostimulant usually comes in the form of substances and/or microorganisms mixtures, aiding the plant in terms of nutrient efficiency and tolerance to biotic and abiotic stresses [8,9]. In addition to the definition, the categorization of biostimulants is somewhat controversial, varying from what each author considers most important [7]. Even though some consider the mode of action to be more relevant, the origin of the biostimulant could provide us with more tools for comparison between products and their effects on plant species. Therefore, the most widely accepted biostimulant categorization comes from du Jardin [6], dividing them into seven categories, namely: humic and fulvic acids; protein hydrolysates and N-containing compounds; seaweed extracts and botanicals; chitosan and other biopolymers; inorganic compounds; and beneficial fungi and bacteria. However, as described by Carletti et al. [10], novel compounds with biostimulant activity are frequently reported in the literature, highlighting the need for a better understanding of their molecular impact and consequently better categorize them.

Results from biostimulant applications vary depending on a range of factors such as the dose used, the mode of application, the timing of application and their composition of one to several biomolecules and/or microorganisms. As such, understanding the influence of biostimulants on plant physiology and molecular pathways therein should be the focus of future studies, in order to elucidate their mechanisms and increase their efficiency [11]. This becomes a difficult task due to their heterogeneous composition. However, recent biostimulant research has made significant progress towards developing this understanding. Previous reviews have comprised information regarding the physiological effects of specific biostimulants categories, with some already considering research surrounding their molecular influence [12,13,14]. However, in this work we focused on various biostimulant categories and the most recent developments surrounding their molecular activity. Thus, focusing on the categorization proposed by du Jardin [6], the objective of this work is to highlight the most recent research surrounding the effects of several biostimulant categories on different crops and how gaining a deeper knowledge of their molecular impacts may lead to an increase in their efficiency.

## 2. Humic Substances as Biostimulants

Humic (HA) and fulvic (FA) acids, or humic substances (HS), have been known to act as biostimulants for some time, with various proven biological activities [15,16]. Consisting of organic compounds from the decomposition of dead biota in soils, HS are highly heterogeneous in their molecule combination and mostly impervious to microbial decomposition [15,17,18,19]. Several roles have been attributed to these substances in regards to soil and plant functions [20]. When it comes to plants, the influence of HS in growth can be indirect, ranging from an increase in microbial populations, improved cation exchange capacity and pH buffering properties of the soil, increased availability and mobilization of soil nutrients and the improvement of soil structure [21,22,23,24]; or direct, by positively influencing several molecular mechanisms such as photosynthetic activity, protein synthesis and enzymatic activity, whilst also being able to effect phytohormones [25,26,27]. Even though HS are promising biostimulants, there is still a need for further research regarding their effects and mode(s) of action [22,28].

### 2.1. Humic Acids

Even though primarily associated with the enhancement of root growth [29], HA have also been shown to confer other benefits such as increasing mobilization of nutrients, improving photosynthetic rates, respiration and water balance and increasing the content in photosynthetic pigments [30,31]. HA are comprised of amino acids, polysaccharide residues and aromatic and aliphatic compounds, with the functional groups of aromatic rings, carboxyl (R-COOH) and hydroxyl (R-OH) seemingly playing an important role in plant nutrition by forming complexes with the cations of essential nutrients [17,28,32,33]. Hormone-like behavior has also been attributed to HA, with the most common being due to the presence of auxin [34], molecules mimicking the same function [35,36] or by interacting with plant hormone signaling pathways [37]. However, HA were also found to increase peroxidase (POD) expression, which has been reported to be involved in auxin metabolism [38]. In recent literature, foliar application of HA has been shown to improve yield in different *Brassica napus* genotypes [39]. Moreover, these authors observed an increase in chlorophyll content, which might be linked to an increase in photosynthetic rate and in RuBisCO activity [40,41,42]. However, previous studies observed some HA not to contain any major components, being comprised of mostly C,H and O [43]. Nonetheless, in this study the application of HA affected the expression of one thousand genes, influencing almost all metabolic pathways including photosynthesis, cell metabolism and phytohormones [43]. In fact, another study using a proteomic analysis in *A. thaliana* roots treated with earthworm feces extracts concluded that 92 proteins were differently expressed in the exposed plants [44]. Further analysis using bioinformatic tools grouped these proteins in three major clusters according to their biological function: protein synthesis; protein folding and elongation; and energy and metabolism. Moreover, these authors were also able to relate the identified proteins to different biological processes, namely: cell wall and energy metabolism, respiration, protein synthesis, protein folding, protein degradation, response to inorganic substances and heat and cell trafficking and division. These results can very well path the way towards a better understating of the molecular pathways positively affected by HA. A metabolomic study also using *A. thaliana* and treatments with HS as a biostimulant observed significant reductions in the concentration of carbohydrates and most of the free amino acids in the roots [45]. These authors also denoted an increase in protein content in the leaves and roots, probably due to higher metabolic activity and protein synthesis, which could be supporting the higher growth rate in plants treated with HA. Byun et al. [46] studied the impact of applying HA from different soil sources on three species of moss, concluding that HA have positive effects on growth and photosynthetic efficiency. These results were largely dependent on the species subject to the treatment, as higher concentrations also impaired the overall growth in some.

Drought stress is one of the most impairing stresses affecting crop growth and productivity, whilst also reducing metabolic and enzymatic activity in plants [47,48,49]. Under water deficit conditions, the addition of HA to the soil was shown to mitigate the negative effects in plants by improving the production of photosynthetic pigments while keeping the relative water content at higher levels, thus enhancing photosynthesis [50,51,52,53]. In fact, a more in-depth study on the effects of HA in the photosynthetic mechanism of *Brassica napus* under water deficit revealed that treatment with HA may positively affect the rate of the electron transporter chain, thus exhibiting higher net photosynthesis rates [54]. Gene expression in maize (*Zea mays*) was also observed to be altered in the presence of HA, especially the genes for PM-H^+^-ATPase (*Mh1*), which is fundamental in the electrochemical gradient of cell membranes leading to a better absorption of nutrients [55]; aquaporin 1 (*PIP1*) [56], which aids with the movement of water and solutes at the molecular level; and nitrate transporters (*Nrt2.1* and *Nrt1.1*) [57]. Other studies also showed that treatment with HA may influence the expression of Heat-shock proteins (HSPs) [58], chaperones in the protection of degradation of proteins [59]. The influence of HA in the expression of genes related to water and nutrient movement could very well be the bridge in the understanding of the positive effects that HA have in the mitigation of stress, including their recent association with the protection of DNA [60]. However, more research needs to be carried out regarding this subject.

### 2.2. Fulvic Acids

Similar to HA, FA are constituted by high amounts of carboxylic groups (COOH), while also having high amounts of phenolic compounds and low amounts of aromatic structures [29]. While most literature to date describes the effects of FA when used in combination with HA or seaweed extracts (SWE), some studies have examined the effects of FA alone on plant growth. For example, FA biostimulants were found to improve germination in spring wheat (*Triticum aestivum*), barley (*Hordeum vulgare*) and sugar beet (*Beta vulgaris*), whilst also increasing the length of shoots and dry weight of shoots and roots [61]. The same study reported an improvement in grain quality and yield in spring wheat, as well as overall yield in sugar beet. The low molecular weight of FA enables them to penetrate through the pores of membranes, and by forming complexes with cations could lead to the transport of nutrients into the cell [29,32,62]. Furthermore, FA have also been observed to promote transcriptional changes in the roots of *Medicago sativa*, up-regulating genes related to biological processes of N metabolism, nutrient transporters and hydrolases [63]. Other studies observed an increase in lipid content correlated with use of FA, as these substances seemingly up-regulated genes associated with lipid biosynthesis [64], as well as genes related to K transporters, starch degradation and plant metabolism [65]. As previously mentioned, abiotic stresses have a high impact on agricultural activity. Similar to HA, FA may also play a potential role as biostimulants in the struggle against abiotic stress. For instance, drought stress leads to a rapid accumulation of reactive oxygen species (ROS) in plant tissue, causing a variety of negative effects at the cellular level [66], which can be attenuated by the function of ascorbic acid. Notably, FA application was shown to improve ascorbate, glutathione and flavonoids, by the upregulation of genes related to their metabolism, ameliorating the negative effects of drought stress [67,68].

The application of HS can improve plant growth parameters, increase the content of photosynthetic pigments, carotenoids, total phenols, flavonoids and NPK concentration [69]. However, as suggested by García et al. [28], there should be a greater emphasis on the study of HS, as they may possess unique properties which might explain their functions.

## 3. Protein Hydrolysates

Food and agricultural industries generate large amounts of organic biomass due to the production and processing procedures involved in manufacturing large quantities of food [70]. This biomass is usually enriched with secondary metabolites, which can be used to produce protein hydrolysates (PHs) through the hydrolysis of raw materials, either of plant or animal origin. Extraction is typically undertaken under acidic, neutral or alkaline conditions and may involve hydrolysis or biological processes through the use of proteolytic enzymes [71]. Depending on the method, these processes extract cellular components or break down proteins, leading to a mixture of free amino acids, polypeptides and oligopeptides, as reviewed by Moreno-Hernández et al. [72]. As the need for sustainability is increasing along with a growing world population, recycling by-products derived from agricultural and industrial activities to obtain PHs and applying them as biostimulants back into the production chain, could be beneficial from both an economical and ecological perspective [6,9,73,74]. 

PHs have been shown to promote plant primary and secondary metabolism [75,76]. The breakdown process in the manufacture of PHs leads to the production of small peptides and amino-acids, which display phytohormone-like activities [77,78]. Other properties of these biostimulants include higher nutrient uptake due to the increase in solubility and mobility of micronutrients, increase in the density, length and number of lateral roots, as well as an increase in enzymatic activity [74,77,79,80]. Recently, foliar application of PH biostimulants was demonstrated to promote the growth of epiphytic bacteria, plant growth and productivity [81]. Commercial PHs (Sinergon Bio) of animal origin applied to olive tree (*Olea europaea*) were associated with positive effects on plant growth and increased photosynthetic rate [82]. Similarly, PHs application in *Diplotaxis tenuifolia* L. also led to an increase in plant dry weight, improved efficiency in chlorophyll biosynthesis and increased activity of the photosynthetic system [83]. The authors also reported an increase in nutrients such as sodium, nitrate, magnesium, potassium, calcium and phosphate, when applied in combination with the microorganism *Trichoderma Harzianum* T22. The increase in photosynthetic rate is most likely related to the direct action of PH, as there is no accumulation of intercellular CO_2_ despite a higher stomatal conductance, suggesting a direct influence of the biostimulant. In fact, other reports of foliar application of PHs demonstrated an amelioration in the gas exchange and transpiration rates, as well as an increase in photosynthetic rate and stomatal conductance [84].

### The Molecular Influence of Protein Hydrolysates

In terms of molecular influence, some studies have attempted to uncover the influence of PHs on gene expression under normal and stress conditions. *Alfalfa*-based PHs were shown to cause up-regulation of genes related to photosynthesis, nutrient uptake and primary metabolism [85]. Additionally, several authors observed a positive effect in the regulation of key genes associated with nitrate and ammonia transporters, as well as nitrate reductase which aids in the conversion of N into amino acids [86,87,88]. Furthermore, under stress conditions, foliar application of PHs has been shown to activate defense response mechanisms in *Arabidopsis thaliana* and *Cucumis sativus* L., more specifically by inducing defense response genes such as *CAT3* and *OXI1*, both related to protection against oxidative stress [89,90], and *PDH*, *GSTF7* and *PR1*, which are related to biotic stresses [75,91,92]. Moreover, foliar and drench application of PHs on tomato under drought stress was observed to completely alter the metabolome in comparison to untreated plants, improving the response to this stress [93]. PHs also seem to play a role in the mechanisms underlying phytohormonal response to stress [78]. Recently, Casadesús et al. [94] studied the application of an animal-based protein hydrolysate (Pepton) in tomato under water stress, observing a significant increase in the hormone profile in these plants. Auxin, cytokinin and gibberellin concentrations were increased in treated plants, aiding plant growth under drought stress conditions. In fact, these changes could be associated with the expression of genes involved in the metabolism, transport and signal transduction of phytohormones, produced by PHs [95]. Another study with the application of a commercial legume-derived PH biostimulant (Trainer^®^) in tomato observed a positive influence in root development [96]. Furthermore, by performing a metabolomic analysis, these authors evidenced an increase in phenylpropanoids, terpenes, flavonoids, nitrogen-containing compounds, glucosinates and alkaloids, while also observing auxin-like activity. Different concentrations of a PH-based biostimulant applied to maize roots has also been shown to alter the plant’s transcriptome and proteome [97]. These authors observed differences in the expression of 1006 genes, as well as 242 differentially abundant proteins. Moreover, most of these genes and proteins were related to metabolic pathways, ROS-related systems, phytohormones, transport and cytoskeletal reorganization, extremely important processes for both plant growth and development, and plant stress tolerance.

Both the constitution and metabolic engagement of PHs have been associated with the observed effects in plant growth and stress tolerance. As more research regarding the molecular effects of these substances begins to surface, the scientific community will gain a more thorough understanding of the effects and potential of this kind of biostimulant in agricultural activity. However, similar to other products, there is a need for more research, as effects vary between different plant species.

## 4. Seaweed Extracts

Seaweeds, as commonly known, are macroscopic photoautotrophic marine algae. These multicellular organisms are significant producers of biomass in marine habitats and represent an excellent economic and renewable resource with several potential uses [98]. Deemed one of the most studied types of biostimulants, seaweed extracts (SWE) are obtained through a variety of processes: alkali, neutral or acid extractions, processes consisting of the disruption of the seaweed by milling under high or low pressure, with an after addition of an acid, alkali or water; rupture of the cells through low temperatures and high pressure; and crushing of frozen seaweeds in order to obtain a suspension of fine particles [98]. The use of seaweeds in society is well established, with their use dating back to ancient times [99]. The relative notoriety of SWE usage in agriculture stems from the positive influence of SWE on plant growth, yield, nutritional quality and their bioactive content [100]. Furthermore, the application of SWE has also been associated with an increase in plant tolerance to biotic and abiotic stresses [100,101,102]. In recent years, there has been a growing interest in SWE amongst the scientific community and the agricultural industry, as several studies indicate a positive influence of SWE in crop production in both normal and stress conditions [103,104,105]. However, it has been shown that the efficacy of seaweed extracts may depend on whether or not a stress is present or absent, the type of stress involved, the type of extract and it biochemical characteristics [101]. Several macroscopic algae are used in the production of biostimulants, although in recent times, particular attention has been placed on the species *Ascophyllum nodosum, Ecklonia maxima* and *Kappaphycus alvarezii*, as well as the genera *Gracilaria* spp. [106].

For many years, the mode of action of seaweed extracts has been investigated by means of bioassay. These studies suggested that application of certain SWE can improve plant shoot and root growth, potentially analogous to growth effects obtained by exogenous application of synthetic growth hormones. SWE were therefore widely described as having “growth hormone-like activity”, with some studies hypothesizing that such effects may be direct in nature and due to the presence of growth hormones detected in certain seaweed extracts [107,108,109,110,111,112,113,114,115,116,117,118,119,120,121,122,123,124,125]. The presence of growth hormones or growth hormone-like substances in SWE may originate from endogenous production of phytohormones by certain species of seaweed during their living phase [109,126,127]. However, these hypotheses have not been validated at the molecular level and it is now recognized that hormone concentrations in SWE are at levels too low to invoke physiological responses in plants, particularly given the low application rates applied at field level [128,129]. Studies indicate that growth hormones in SWE may be at low nanogram or picogram per mL levels of extract [123] or at undetectable levels which suggests their potential absence from certain extracts [128]. While some studies suggest that application of SWE may increase plant growth or modulate the expression and localization of growth hormones within plants [130,131], it has not been demonstrated that such effects are a due to the presence of hormones. As such, the “growth hormone model” of how SWEs influence plant growth is not fully supported by the literature and other mechanisms must therefore be considered. It has been suggested that non-growth hormone components in seaweed, such as polysaccharides, may be responsible for growth enhancing effects induced by SWE [132,133]. Polysaccharides are major components of brown seaweeds [134], and recent studies show that a particular commercial *Ascophyllum nodosum* (Super Fifty) extract high in polysaccharides modulates a range of processes at the transcriptomic, metabolic and lipid levels [135,136,137]. The authors demonstrate that these changes involve multiple pathways and culminate in significant changes at the phenotypic level, including: tolerance to oxidative stress and abiotic stresses; reductions in Reactive Oxygen Species (ROS); reductions in electrolyte leakage and increases in plant growth. As such, new studies are beginning to change our understanding of the modes of action of SWE and shifting the focus to specific polysaccharides and other non-growth hormone molecules as the most likely drivers of effects observed in plants.

### 4.1. Ascophyllum nodosum

Considered a brown alga, *Ascophyllum nodosum* is one of the most studied macroscopic algae [132], and is used in a variety of available products due to its high polysaccharide and phenolic content [102,132]. Even though some of these extracts are already commercialized for agricultural purposes [138], and even applied in the cultivation of other algae [139], research in past decades was focused primarily on the general benefits of these extracts. However, the focus has been shifting towards a deeper understanding of the molecular influence these extracts could have, especially in the fight against abiotic stress. Increases in plant growth and yield parameters by application of *A. nodosum* biostimulants is extensively reported in the literature. More recently, studies regarding the effects in spinach demonstrated this SWE induced an increase of up to 50% of fresh yield, increase protein and nutrient content, and the concentration of phenolic compounds in the leaves [140]. Another study in grapevine also demonstrated an increase in yield, N concentration and anthocyanins, without negatively affecting the quality of the berries [141]. It has been reported that grape berry quality was increased following application of *A*. *nodosum* SWE. The improvements in anthocyanin accumulation observed in this study may provide a means of producing premium wines [142]. Increases in fruit quality after the application of these SWE has also been observed in sweet cherry (*Prunus avium* L.), leading to larger fruits, increased content in soluble solids, polyphenols, vitamin C and antioxidants, as well as improving fruit color, acidity, ripening timing and reduced cracking [143,144]. Interestingly, the application of *A. nodosum*-based biostimulants has been observed to lead to changes in the expression of cherry cell-wall and cuticular wax genes (*PaEXP1*, *Pa⊎-Gal* and *PaWS*), which can be correlated to a reduction in fruit cracking [145]. New research regarding effects on flowering and fruit setting in eggplant also opened up a possible new function for these biostimulants, as these characteristics were positively influenced with the application of this SWE [146]. 

The true potential of *A. nodosum* SWE is tied to stress tolerance in several species. Under limited phosphorus conditions, SWE of *A. nodosum* was shown to improve the growth of *Zea mays*, increasing overall biomass, NPK content and photosynthetic pigments when compared to the control [147]. Moreover, application of this biostimulant led to a reduction in oxidative damage and electrolyte leakage, whilst increasing the total content of soluble sugar, phenolic compounds, flavonoids and amino acids. Interestingly, these results were correlated to positive changes in gene expression affecting the complex mechanism of P homeostasis of *Zea mays* [147]. Effects on the improvement of thermo tolerance in tomato has also been observed recently, especially in the pollen viability and chlorophyll levels [148]. The same authors also denoted a positive effect in the synthesis of HSP, promoting a better heat stress tolerance in these plants. Amelioration of the effects of drought stress is also one of the attributed functions to *A. nodosum* SWE. Recently, soybean submitted to water stress and treated with this biostimulant was able to restore its’ water content while also promoting the growth of the root system, increase photosynthetic efficiency and, chlorophyll content [149]. Additionally, *A. nodosum* SWE was shown to improve the response of *Corylus avellana* trees to heat and drought stress, while preserving the quality of the kernel [150,151]. In fact, nut and kernel biometric parameters increased in comparison to control, alongside the concentration in vitamin E, phenolics and antioxidant activity. 

While several studies demonstrate the general positive effects of *A. nodosum* extracts, it is also recognized that the efficacy of these biostimulants is dependent on several variables. In particular, a significant level of specificity has been shown for seaweed extracts derived from *A. nodosum* in enhancing plant growth and tolerance to stress, whereby the underlying extraction method employed is strongly associated with the stress tolerance effects observed [101]. As such, commercial extracts of *A. nodosum* are likely to confer differential effects depending on how they are manufactured, their constituents, their bioactive composition, bioactive levels and the plant stress types involved. Consistent with this, it has been shown that different commercial extracts of *A. nodosum* can induce differential effects. In a recent study, tomato plants treated with *A. nodosum* based biostimulants, SuperFifty and Rygex, showed significant differences in terms remodeling leaf nitrogen metabolism and accumulation of minerals such as nitrate and magnesium in the leaf under normal and salt/and or osmotic stress conditions [152]. 

Molecular priming using SWE is a promising tool in the battle against abiotic stress [153]. Abiotic stress events such as cold, drought, heat and pollutants lead to harmful accumulation of ROS in plants. The accumulation of ROS at levels that incur damage is referred to as oxidative stress, which can bring irreversible damage to cellular components and can compromise plant growth and yield. Priming with a commercial extract of *A. nodosum* (SuperFifty) has been shown to inhibit ROS production, and protects the model plant *Arabidopsis thaliana* and crops, tomato and pepper, from severe oxidative stress [135,136]. Priming and foliar application with SuperFifty led to a better tolerance against drought stress in *Arabidopsis thaliana*, with primed plants showing better development than control plants. Moreover, electrolyte leakage was observed to decrease in treated plants, and the reduction in relative water content (RWC) and ROS accumulation due to drought were shown to be diminished [137]. More interestingly, plants primed with this SWE displayed a substantial amount of differently expressed genes, suppressing those with negative effects such as ROS accumulation and upregulating those with positive effects such as ROS scavengers. Stress responsive negative regulator of growth, *RESPONSIVE TO DESICCATION 26* (*RD 26*) was repressed and cell cycle genes were activated in shoot apical meristems of SWE primed plants, revealing an active cell division and growth taking place in these plants during drought [137]. Priming with *A. nodosum* extracts (SuperFifty and Rygex) also induced pre-adaptive physiological responses, improved yield and reallocated the biomass towards the fruits in tomato plants during salt stress [154]. Overall, the model emerging from these recent studies is that certain *A. nodosum* extracts can induce molecular priming and can activate a wide range of molecular changes, which manifest at the phenotypic level, culminating in enhanced tolerance to oxidative and abiotic stresses. Moreover, the replication of these effects in both model and crop plants indicates that the stress tolerance pathways modulated during priming may be shared across multiple crop species. 

### 4.2. Ecklonia maxima

*Ecklonia maxima* is also considered a brown alga, and similar to *A. nodosum* also has interesting properties when used as a SWE biostimulant, potentially due to compounds present in these extracts which may include, amino acids, nutrients, alginates and phytohormones [98]. In recent studies, application of a commercial SWE of *E. maxima* (Kelpak) in common bean led to an increase in yield and antioxidant potential, as the concentration of phenolics, flavonoid and anthocyanins was higher [155]. The same product also produced similar positive effects in spinach, while improving the concentration of chlorophylls, carotenoids, proteins and phytohormones [156]. Moreover, under sub-optimal N concentration in the soil, foliar application of *E. maxima* improved the same parameters in baby leaf lettuce [157]. Positive effects of this SWE on stress mitigation have also been recently reported. For instance, while saline conditions can reduce yield and produce quality, it has been demonstrated that foliar application of *E. maxima* in zucchini squash (*Cucurbita pepo* L.) can mitigate these effects [158]. The authors reported higher yield, biomass and fruit quality in comparison to untreated plants, whilst also observing an improvement in SPAD index and photosynthetic synthesis. Despite the recent research on this SWE, we could not find reports on the molecular influence it may have.

### 4.3. Kappaphycus alvarezii and Gracilaria edulis

Being a low cost and fast growing red alga, *Kappaphycus alvarezii* is widely cultivated due to being edible as well as being a source of carrageenan [159,160]. However, several other uses have been assigned to this seaweed due to its’ constitution and potential to be applied in a variety of commercial products [161]. Despite variability in extraction processes, these SWEs have been shown to contain nutrients, hormones and several other compounds. These extracts have also been extensively studied, largely due to their potential to improve crop production, plant growth and mitigate the effects of abiotic stress [162]. Similar to other biostimulants, recent studies on the application of *K. alvarezii* SWE have demonstrated positive results in the improvement of plant growth and yield in species such as sugarcane [163], maize [164,165,166], rice [167,168] and potato [169,170]. These effects are likely associated with the chemical composition of these SWEs. In fact, recent studies show that application of extracts of *K. alvarezii* and *Gracilaria edulis* improve germination parameters in rice (*Oryza sativa*) seeds, with foliar application of these biostimulants leading to the improvement of plant growth and biomass [168]. The most interesting observation was the increase of up to 15% in yield, as well as the content in nutrients such as N, P, Zn, Cu, Fe, Mn and K. Seaweed extracts of both *Kamaphycus* spp. and *Gracilaria* spp. have been shown to contain glycine betaine and choline, as well as plant growth regulators such as indole-3-acetic acid (IAA), zeatin, gibberellic acid (GA_3_) and several macro- and micronutrients, which may potentially explain these results [162]. 

Special attention has been given to these SWEs in the mitigation of the effects of abiotic stress. For instance, under salt and/or drought stress, the use of *K. alavarezii* extracts in *Triticum durum* was shown to enhance plant growth, increase root growth, photosynthetic pigments content and RWC, whilst also presenting a higher accumulation of osmoprotectants such as proline, amino acids and soluble sugars, conferring plant stress tolerance [171]. Moreover, while evaluating the molecular influence of this SWE, *Triticum* spp. stress responsive genes such as *WCK-1*, *TaWRKY10, TdCAT* and *TdSOD* were upregulated, indicating a direct influence in the gene expression. In fact, similar results were observed in *Zea mays*, where besides those related to oxidative stress, overexpression of transcripts for fatty acid metabolism, starch synthesis, nutrient transport and metabolism, as well as cell cycle and division was also observed [172]. Despite being observed in plants under stress, this molecular influence may potentially explain some of the positive effects observed at the macroscopic level in more general studies. Once again, this highlights the need for more research at the molecular level, in order to achieve a greater understanding the mode(s) of action involved.

## 5. Microorganism-Based Biostimulants

Even though some authors classify microorganism inoculates as being biofertilizers [173,174,175,176], these could very well be referred to as biostimulants [7]. Fungi and bacteria-based biostimulants may have a role to play in mitigating the impacts of agricultural activity on the environment [177], such as positively influencing the soil biodiversity [178]. Moreover, microorganisms play a key role in the phyllosphere, rhizosphere and endosphere of plants increasing the availability of certain nutrients and facilitating their absorption, with the symbiosis between both being a key factor in their evolution [179]. Most microorganisms which directly or indirectly interact with plants are denominated Plant Growth Promoting Bacteria (PGPB), which includes both free living bacteria in the soil as well as rhizobacteria which colonize the rhizosphere [180,181,182]. Several functions are credited to these microorganisms, including the synthesis of plant growth regulators and the solubilization of inorganic nutrients [183]. PGPB species, such as *Arthrobacter* spp., *Pseudomonas* spp., *Rhodococcus* spp., *Enterobacter* spp., *Ochrobactrum* spp., *Acinetobacter* spp., *Bacillus* spp., *Rhizobium* spp., *Streptomyces* spp. have been actively studied to investigate their potential role as biostimulants, with some already being commercialized [16,184,185,186].

### 5.1. PGPB as Biostimulants

In the case of *Bacillus* spp., these bacteria not only act as biofungicides promoting plant and soil health [187,188,189], but also as biostimulants due to the metabolites produced and the solubilization of essential nutrients to simpler forms for root uptake [190]. These microorganisms have also been associated with the production of growth promoting substances such as cytokinins, spermidines, gibberellins and IAA [190]. In recent studies, *B. pumillus* was observed to increase nutrient content in fruit and fruit yield in tomato (*Solanum lycopersicum* L.), and when in combination with *P. putida* there was an increase in healthy fruit yield. [191]. Three *B. velezensis* strains were also studied in wheat (*Triticum aestivum* L.) where it positively affected early development, while increasing the concentration of macro- and micronutrients in the plant under greenhouse conditions [192]. Moreover, the same authors found an increase in wheat grain yield grown under low N content when inoculated with *B. velezensis* FZB24. In fact, while limited N availability in soil can impair plant growth, some *Bacillus* spp. strains are able to produce it from atmospheric N_2_ leading to higher yield and plant growth enhancement [193,194]. Moreover, Nguey et al. [195] first reported that *B. megaterium* SNji can mitigate the negative effects on root growth in wheat caused by high N concentrations in the soil, which could be due to the use of N by the bacteria itself [196]. This duality of functions of *Bacillus* strains opens the possibility for multiple uses, as both low or high concentrations of N in the soil affect plant growth and yield. *Bacillus* has also been associated with the synthesis of IAA, cytokinins, gibberellins and spermidines, which promote plant growth [197,198]. Soybean under salt stress was shown to have increased gibberellin and abscisic acid concentrations when *B*. *amyloquefaciens* was present [198]. In fact, strains such as *B*. *amyloquefaciens* SQR9 were shown to secrete IAA and GA_3_ which improved root growth of maize under salt stress, while also positively affecting the expression of RuBisCO *rbcS* and *rbcL* genes, key enzymes on photosynthesis [199]. Upregulation of the *NHX1*, *NHX7*, *H+-PPase* and *HKT1* genes was also observed, indicating an active role of *B. amyloquefaciens* in the sequestration of Na^+^.

Another microorganism of high relevance is *Pseudomonas* spp. Several strains have been associated with biostimulant activity, including enhancement of plant nutrient uptake, vitamin secretion and synthesis of aminocyclopropane-1-carboxylate (ACC) deaminase [200,201,202]. In recent studies, *P. fluorescens* LBUM677 was shown to increase seed weight and number, as well as the oil content in *Brassica napus*, *Buglossoides arvensis* and *Glycine max*, which was attributed to its’ production of ACC deaminase, IAA and solubilization of micronutrients [203]. Recently, *P. fluorescens* was also shown to increase Ca, Mg, K, P and Zn concentration in *Amaranthus hybridus* L. leaves, positively affecting its’ nutritional quality despite impairing plant growth [204]. Results on growth impairments should be interpreted with caution, as the use of high concentrations or certain combinations of biostimulants could be potentially toxic. Application *of P. pseudoalcaligenes* and *P. putida* was also shown to increase water content and photosynthetic pigments, positively affecting plant growth [205]. Moreover, the same authors performed a salinity stress assay, with the plants treated with these microorganisms performing better than the control. Similar results were observed in *A*. *thaliana*, in which the inoculation with *P. koreensis* Ps 9–14 led to an amelioration of the salt-toxicity effects in plant growth, most likely tied to the increased antioxidant activity of APX, CAT and POD [206].

Primarily associated with their symbiotic relationships with legumes, *Rhizobium* spp. are notable for their ability in reducing atmospheric nitrogen, solubilization of nutrients, production of secondary metabolites and plant growth hormones [207]. The application of these PGPB as biostimulants has increased in the recent years. In particular, studies on the inoculation of chickpea with *Rhizobium* sp. significantly enhanced plant biomass and yield, and when combined with foliar application of GA_3_ led to increases of up to 39% [208]. Furthermore, higher chlorophyll content and NPK content was observed, with positive effects in the nutritional content of chickpea seeds observed. Despite the application of GA_3_, rhizobia have the capacity to synthesize hormones such as gibberellins and IAA. In fact, *Rhizobium radiobacter* InaCCB835 not only led to an increase in plant biomass, number of leaves and root length in *Brassica rapa* L., but also increased the total content of IAA, P and N in the plant [209]. Similar results were obtained in other studies, once again justifying the positive effects *Rhizobacterium* spp. could have on non-legume species [210], even under saline conditions [211]. In fact, a recent study of the application of *Rhizobium jaguaris* CCGE525 inoculates applied to *A. thaliana* reported increases in plant growth under normal conditions, leading to higher biomass and higher chlorophyll content in the leaves [206]. The same authors studied the potential of this strain in the amelioration of salinity-stress toxicity, observing a better response of the plant, induction of physiological and biochemical responses, whilst also increasing proline content which can act as an osmoprotectant. Despite the positive results observed throughout the literature, the influence of *Rhizobium* spp. on gene expression is usually associated with their relationship at the root level, primarily by increasing expression of *nod* genes which leads to higher nodulation [212].

The study of the biostimulant activity of *Arthrobacter* spp. has increased in recent years. For example, studies involving the use of *Arthrobacter agilis* UMCV2 inoculates in strawberry demonstrated an increase in yield, with volatile compounds produced by this strain almost doubling the germination of achenes, which could potentially ameliorate the low germination rate of strawberries [213]. Research on the application of this microorganism in *Sorghum bicolor* demonstrated a promotion in plant growth and chlorophyll accumulation possibly due to higher Fe acquisition [214]. Interestingly, genes related to iron absorption and transport, *IRT1*, *IRT2*, *YS1* and *YS2*, were upregulated, indicating an influence on gene expression. 

### 5.2. Trichoderma spp.

When it comes to fungi, one of the most promising species belongs to the *Trichoderma* genus [215]. Even though typically associated with biopesticides, several *Trichoderma* spp. strains have been gaining increasing interest as biostimulants due to their abilities to improve tolerance to abiotic stresses and increase plant growth, development and yield [216,217,218]. Recently, Visconti et al. [219] studied the effect of *Trichoderma virens* GV41-based biostimulants in both lettuce and rocket, observing an increase in phenol content and antioxidant activity and improved nitrogen usage efficiency in lettuce, suggesting their potential application in the management of soil N fertility. Studies using *Trichoderma* strains and their bioactive metabolites, either alone or combined, reported an increase in plant growth in soybean, as well as in fatty acid and mineral content in their seeds [220]. *Trichoderma harzianum* T22 has also been shown to have biostimulant properties in wheat, enhancing spike fresh weight and shoot dry weight under normal conditions, while increasing the number of stems, dry weight and spike fresh weight under water stress [221]. The same authors also found this *Trichoderma* strain was associated with increases wheat biomass under low N availability conditions, which might indicate an increase in stress tolerance. Similarly, other authors observed an increase in N uptake and yield in lettuce with the use *T. virens* GV 41 biostimulants [222]. *T. saturnisporum* has also been shown to enhance germination, increase plant vigor and yield, whilst also leading to better fruit quality in melon [223]. A recent and comprehensive study on the influence of *Trichoderma* strains in strawberry, described very interesting findings obtained from proteomic analysis [224]. Aside from the common conclusions of increases in biomass, yield, nutrient uptake, anthocyanins and antioxidants content, the proteomic analysis showed augmented levels of proteins involved in carbohydrate metabolism, glycolysis and alcoholic fermentation; higher concentration of components of the NADH dehydrogenase complex and biosynthetic machinery; as well as defense-related and vesicle machinery components [224]. This work reflects the importance of understating the molecular effects of biostimulants, as it opens way for the fine tuning of biostimulant application in plants.

Some authors also report positive effects of *Trichoderma* strains in plants under stress conditions. More recently, the application of *T. ligibrachiatum* to *Healianthus annus* L. under lead stress conditions was associated with a positive effect in its’ antioxidant activity when compared to the control [225]. This could be due to certain *Trichoderma* strains being able to influence the bioavailability of lead in the soil by releasing chelators [226]. Cold stress effects in tomato plants were also observed to be mitigated by *T. harzianium* AK20G, despite individually impairing it [227]. These authors noted an increase in biomass, RWC and photosynthetic rate. Moreover, the observed decrease in electrolyte leakage was possibly due to a higher expression of the *P5CS* gene, which encodes for delta 1-Pyrroline-5-Carboxylate Synthetase, a key enzyme involved in proline synthesis, leading to the accumulation of this stress protective osmolyte in plants. In fact, higher proline accumulation with the application of *Trichoderma* spp. inoculates has also been observed in wheat under salinity-stress, leading to a positive effects in the photosynthetic performance of these plants [228]. 

Despite the clear effects of PGPB and *Trichoderma* spp. as biostimulants, improving both quality parameters as well as stress-tolerance, most of the mechanisms underlying these results remain undiscovered.

## 6. Conclusions and Future Directions

**Figure 1:**
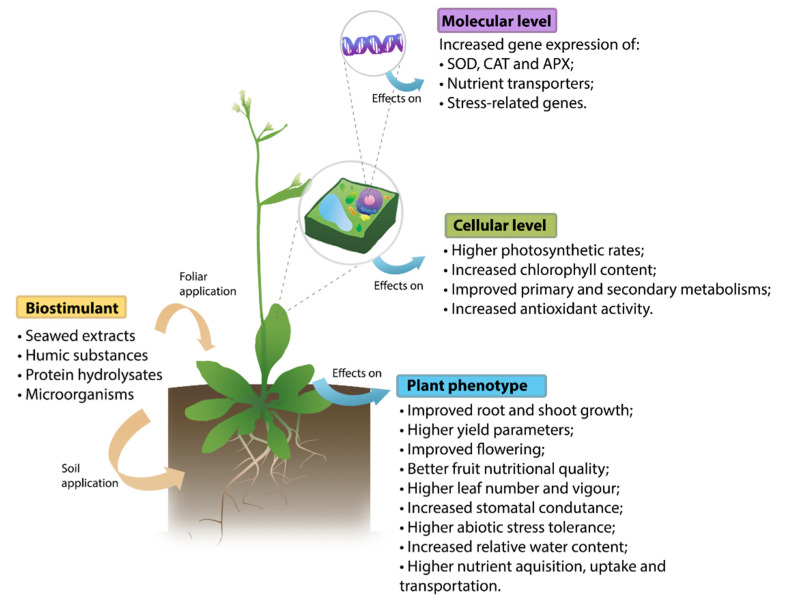
Summarized representation of the effects of biostimulants in plants at the molecular and cellular level, and in the plant phenotype.

The use of plant biostimulants as substitutes for more conventional methods in agriculture appears to be growing in prominence, with more and more research shedding a light on how effective they can be. Research ranging from a variety of different plants (Table 1) demonstrates that biostimulants have the capacity to improve plant growth and development; increase nutrient uptake, yield and water content, whilst also improving the nutritional value and quality of their produce (Figure 1). 

Moreover, with the unavoidable effects of abiotic stress due to soil pollution as well as climate change, biostimulants may provide a solution to ameliorate their effects in the agronomic industry. Despite this, we still need to consider a number of factors: effects can differ between crop species, extraction/production processes for biostimulants and their levels of constituents, bioactives and effects can vary and distinct biostimulants can act differently in the same species. As such, the increasing knowledge at the molecular level, mostly the influence in gene expression, can open an array of possibilities for the fine tuning of these products. As expressed throughout this review, some research has been undertaken to further this understanding, with some molecular pathways and alterations in the expression of genes already being uncovered. However, this matter still has a long way to go and further research must be carried out. Moreover, as molecular pathways triggered by biostimulants become identified, it will be important to conduct functional work to fully elucidate the precise mode(s) of action employed, in a range of model and crop plants. Such research must also take cognizance of the different commercial products involved, the extraction methods and the underlying composition of these extracts. With the collective collaboration between the scientific community, the potential of these products to enhance agricultural sustainability and increase food security in the face of climate change, may be realized.

## Figures and Tables

**Table 1:** Summarized response of different plant species to biostimulants.

Biostimulant	Plant Species	Plant Response	References
Humic acids	*Achillea millefolium* L.	Improved growth parameters: increased photosynthetic pigments, total phenols, total flavonoids and antioxidant activity of the leaves and flowers were increased significantly.	[69]
	*Arabidopsis thaliana*	Enhanced thermotolerance by upregulation of heat-shock protein genes under heat stress; Increased concentrations of proteins related to cell wall and energy metabolism, respiration, protein synthesis, protein folding, protein degradation, response to inorganic substances and heat and cell trafficking and division; decreased concentration of carbohydrates and amino acids.	[44,45,58]
	*Brassica napus*	Increase in yield, chlorophyll content; improved oil quality, plant net photosynthesis, gas exchange rate and electron transport flux; decrease in soluble carbohydrates, linolenic and erucic acid.	[39,54]
	*Capsicum annuum* L.	Improved root development and increased plant biomass under drought stress while rapidly decreased leaf stomatal conductance and transpiration rates; increased chlorophyll content leading to improved net photosynthesis.	[51]
	*Echinacea purpurea* L.	Improved plant growth under drought stress; increased flavonoid, phenolic and proline concentration; increased relative water content and photosynthetic pigments concentration.	[50]
	*Hordeum vulgare* L.	Increase in photosynthetic pigment concentration and NPK levels; improved plant growth and yield parameters under drought stress.	[52]
	*Phaseolus vulgaris* L.	Protective effects against DNA hypomethylation and damage; alterations in the expression of stress-related genes.	[60]
	*Rhododendron*	Increase in phenolic content, flavonoids, soluble carbohydrates, starch and soluble proteins; upregulation of peroxidase genes (*POD1*).	[38]
	*Solanum tuberosum*	Increase in tuber yield and plant biomass; improved plant growth, nutrient transport and photosynthetic parameters under drought stress.	[53]
	*Zea mays*	Improved water and nitrogen efficiency; upregulation of genes related to water transport, nutrient absorption and nitrate transporters.	[57]
Fulvic acids	*Achillea millefolium* L.	Improved growth parameters: increased photosynthetic pigments, total phenols, total flavonoids and antioxidant activity of the leaves and flowers were increased significantly.	[69]
	*Beta vulgaris*	Improved germination parameters; increased root size, yield and soluble sugar content.	[61]
	*Camellia sinensis* L.	Upregulation of genes related to metabolism of ascorbate and glutathione, and biosynthesis of flavonoids improving the antioxidant defense under water stress; increased leaf water content and chlorophyll content; reduction in accumulation of ROS.	[67]
	*Hordeum vulgare*	Improved germination parameters.	[61]
	*Medicago sativa*	Upregulation of genes related to early nodulation signaling, N metabolism, nutrient transporters and hydrolases; increased total yield and plant biomass.	[63]
	*Monoraphidium* sp.	Upregulation of lipid biosynthesis genes; increased protein concentration and chlorophyll content.	[64]
	*Paeonia ostii*	Amelioration of drought stress effects; increased plants’ RWC; increased activity of antioxidant enzymes (SOD, CAT and POD) leading to lower ROS concentration; increased photosynthetic parameters; maintained the integrity of mesophyll cell ultrastructure and chloroplasts; increased expression of drought-tolerance genes.	[68]
	*Triticum aestivum* L.	Improved germination parameters; increased yield and grain quality.	[61]
Protein hydrolysates	*Brassica oleracea*	Improved photosynthetic rate and stomatal conductance under drought stress; amelioration of the negative effects of stress in gas exchange and transpiration rates.	[84]
	*Diplotaxis tenuifolia* L.	Increased plant biomass, yield and chlorophyll biosynthesis; improved photosynthetic rate and leaf antioxidant activity; increased nutrient and organic acid concentration.	[83]
	*Lactuca sativa* L.	Stimulated the growth of plant growth promoting bacteria leading to increases in leaf chlorophyll and plant biomass.	[81]
	*Olea europaea*	Improved photosynthetic rate and stomatal conductance; increased plant growth and biomass; had a lasting positive effect in the sink/source ratio.	[82]
	*Solanum lycopersicon* L.	Increased plant biomass, chlorophyll and phenolic content and soluble sugars concentration; improved photosynthetic rate, root growth and upregulation of genes related to antioxidant activity, photosynthesis, nutrient uptake and primary metabolisms. Increased phenylpropanoids, terpenes, nitrogen-containing compounds, glucosinates and alkaloids. Under different N regimes, improved photosynthetic rates and N content in leaves; upregulation of genes related to amino acid and N transport. Under drought stress, increased plant biomass, transpiration rates and stomatal conductance; improved redox status of treated plants and tolerance to ROS-mediated oxidative imbalance; increased antioxidant protection, IAA, cytokinins and jasmonic acid concentrations.	[85,87,93,94,96]
	*Zea mays*	Enhanced plant stress response; improved root growth; increased expression of nitrate transporters and ROS response genes; increased transport and root accumulation of nutrients; upregulation of genes involved in nutrient transport, hormone metabolism, transport and cytoskeletal reorganization; induced changes at the transcriptomic and proteomic level.	[88,95,97]
Seaweed extracts (*Ascophyllum nodosum*)	*Arabidopsis thaliana*	Oxidative and drought stress tolerance, reduced accumulation of ROS and cell damage; downregulation of genes related to growth impairment during stress; upregulation of ROS scavengers, cell cycle and cell division genes.	[135,136,137]
	*Corylus avellana*	Increased plant RWC and CO_2_ assimilation; improved plant water use efficiency; reduced electrolyte leakage, membrane lipid peroxidation, antioxidant enzymes and proline content; increased fruit biometric parameters, antioxidant activity vitamin E, soluble sugars and phenolics content.	[150,151]
	*Glycine max* L.	Under drought stress, increased stomatal conductance, photosynthetic activity and efficiency, chlorophyll content and antioxidant activity; improved root growth and photoassimilates production.	[149]
	*Lycopersicon esculentum*	Under normal and high temperatures, improved thermo tolerance, pollen viability and photosynthetic parameters; increased fruit number and chlorophyll content; upregulation and downregulation of HSP genes; in addition, improved fruit yield components under normal and salt stress conditions	[148,154]
	*Prunus avium* L.	Increased plant yield, RWC, photosynthetic pigments, soluble sugars and protein concentration; improved gas exchange and water use efficiency; reduced fruit cracking; increased fruit size, soluble solids content, polyphenols, vitamin C and antioxidant potential; improved fruit quality, acidity, color parameters and ripening process; up-regulation of genes related to cell-wall and cuticular waxes.	[143,144,145]
	*Solanum melongena* L.	Increased the number of pollen tubes and fertilized ovules; improved flowering and fruiting of the plants.	[146]
	*Spinacia oleracea* L.	Increase plant biomass, protein and nutrient content and concentration of phenolic compounds in the leaves; improved chlorophyll synthesis and photosynthetic rate; enhanced nutritional value.	[140]
	*Vitis vinifera* L.	Increased plant biomass, yield, N and soluble sugar concentration; increased berry number, anthocyanins and phenolics concentration, without negatively affecting their quality.	[141,142]
	*Zea mays*	Under limited phosphorus conditions, improved plant growth; increased plant biomass, NPK, photosynthetic pigments, total soluble sugars, phenolic compounds, flavonoids and amino acids content; diminished oxidative damage and electrolyte leakage; positively affected the expression of genes related to P homeostasis.	[147]
Seaweed extracts (*Ecklonia maxima*)	*Cucurbita pepo* L.	Under salt-stress, increased yield and plant biomass; improved fruit quality and nutritional status, photosynthetic parameters and pigment synthesis; decreased oxidative stress.	[158]
	*Lactuca sativa* L.	Under sub-optimal N concentration in the soil, increased yield, chlorophyll and carotenoids content; enhanced photosynthetic parameters and antioxidant activity.	[157]
	*Phaseolus vulgaris* L.	Increased yield and antioxidant activity; increased biosynthesis of phenolics, flavonoids and anthocyanins; improved nutritional quality of the seeds.	[155]
	*Spinacia oleracea* L.	Improved plant growth, yield and nutritional quality; increased concentration of chlorophyll, carotenoids, protein content and phytohormones; promoted activity of enzymes related to compound biosynthesis.	[156]
Seaweed extracts (*Kappaphycus* *alvarezii*)	*Oryza sativa*	Increased yield parameters, grain number, protein and nutrient content in the grain, plant biomass and chlorophyll content; improved germination, seedling vigor and root growth.	[167,168]
	*Saccharum officinarum*	Increased plant yield and brix content of the juice; improved plant growth.	[163]
	*Solanum tuberosum* L.	Improved plant growth parameters; increased yield, yield quality, nutrient concentration and ascorbic acid and soluble sugar content.	[169,170]
	*Triticum durum*	Increased plant growth, root growth, photosynthetic pigments content, RWC, proline, amino acids and soluble sugars content; improved plant stress tolerance; upregulation of stress response genes (*WCK-1, TaWRKY10, TdCAT* and *TdSOD*).	[171]
	*Zea mays*	Under drought stress, increased yield parameters, photosynthetic pigments, antioxidants and grain quality and protein content; decreased photosystem damage and lipid peroxidation. Under optimal conditions, increased yield parameters and quality, nutrient uptake; improved plant growth, antioxidant activity; decreased lipid peroxidation and accumulation of ROS; upregulation of genes related to fatty acid metabolism, starch synthesis, nutrient transport and metabolism, cell cycle and division.	[164,165,166,172]
Microorganisms (Plant growth promoting bacteria)	*Amaranthus hybridus* L.	Increased leaves nutrient concentration increase; improved nutritional quality, plant growth and photosynthetic pigments under certain circumstances.	[204]
	*Arabidopsis thaliana*	Under optimal and salt-stress, improved plant growth and biomass; increased antioxidant activity, and proline and chlorophyll content.	[206]
	*Brassica napus*	Improved plant growth, plant biomass, yield parameters and seed fatty acid concentration.	[203]
	*Brassica rapa* L.	Increased plant biomass, number of leaves and root length, total content of IAA, P and N.	[209]
	*Buglossoides arvensis*	Improved plant growth, plant biomass, yield parameters and seed fatty acid concentration.	[203]
	*Cicer arietinum* L.	Increased plant biomass and yield, chlorophyll and NPK content; improved seeds nutritional content.	[208]
	*Coriandrum sativum*	Under optimal conditions, increased RWC, photosynthetic pigments concentration; improved plant growth. Under salt-stress, improved plant growth; increased RWC and photosynthetic pigments concentration.	[205]
	*Fragaria × ananassa*	Increased plant yield; improved achene germination and germination rate.	[213]
	*Glycine max* L.	Improved plant growth, plant biomass, yield parameters and seed fatty acid concentration. Under salt stress, increased plant biomass and gibberellin and abscisic acid concentrations; improved plant growth and development.	[198,203]
	*Solanum lycopersicum* L.	Increased plant biomass, RWC, healthy fruit yield, fruit micro- and micronutrient content.	[191]
	*Sorghum bicolor*	Increased plant growth and chlorophyll pigments; upregulation of genes related to iron absorption and transport (*IRT1*, *IRT2*, *YS1* and *YS2*)	[214]
	*Triticum aestivum* L.	Improved early plant development and nutrient uptake; increased plant macro- and micronutrients concentration and grain yield. Under low N content in the soil, ameliorated negative effects on root growth and yield parameters.	[192,195]
	*Zea mays*	Under salt stress, improved root growth; increased chlorophyll and soluble sugar content; decreased lipid peroxidation; improved POD and CAT activity; upregulation of RuBisCO, NHX1, NHX7, H+-PPase and HKT1 genes.	[199]
Microorganisms (*Trichoderma* spp.)	*Cucumis melon*	Increased plant vigor, biomass and yield; improved germination and fruit quality.	[223]
	*Eruca sativa* Mill.	Increased plant biomass, yield, phenols content and antioxidant activity; improved nitrogen usage efficiency and uptake.	[219,222]
	*Fragaria* x *ananassa* Duch.	Increased biomass, yield, nutrient uptake, anthocyanins and antioxidants content, concentration of proteins involved in carbohydrate metabolism, glycolysis and alcoholic fermentation, higher concentrations of NADH dehydrogenase components and defense related machinery components.	[224]
	*Glycine max* L.	Increased plant growth and biomass, nutrient uptake and fatty acid and mineral content in the seeds.	[220]
	*Helianthus annus* L.	Under lead stress, increased antioxidant activity; enhanced heavy metal stress tolerance.	[225]
	*Lactuca sativa* L.	Increased plant biomass, yield, phenols content and antioxidant activity; improved nitrogen usage efficiency and uptake.	[219,222]
	*Solanum lycopersicum* L.	Under chilling stress, increased biomass, RWC and proline content; improved photosynthetic rate; decreased lipid peroxidation and electrolyte leakage; upregulation of genes related to osmoregulators and hormone biosynthesis.	[227]
	*Triticum aestivum* L.	Under salt stress, increased plant biomass, proline and IAA content; improved photosynthetic parameters and water use efficiency.	[228]
	*Triticum durum*	Under normal and drought-stress conditions, increased plant growth and biomass, plant yield; upregulation of genes related to drought stress response.	[221]
